# Quality Improvement with Outcome Data in Integrated Obstetric Care Networks: Evaluating Collaboration and Learning Across Organizational Boundaries with an Action Research Approach

**DOI:** 10.5334/ijic.7035

**Published:** 2023-05-26

**Authors:** Anne Louise Depla, Anna W. Kersten, Marije Lamain-de Ruiter, Marielle Jambroes, Arie Franx, Inge M. Evers, Bettine Pluut, Mireille N. Bekker

**Affiliations:** 1 Department of Obstetrics and Gynaecology, Wilhelmina Children’s Hospital, University Medical Centre Utrecht, Utrecht, the Netherlands; 2 Department of Public Health, Julius Centre, University Medical Centre Utrecht, Utrecht, the Netherlands; 3 Department of Obstetrics and Gynaecology, Erasmus MC Sophia, Rotterdam, the Netherlands; 4 Department of Obstetrics and Gynaecology, Meander Medical Centre, Amersfoort, The Netherlands; 5 Erasmus School of Health Policy & Management, Erasmus University Rotterdam, Rotterdam, Netherlands

**Keywords:** interprofessional learning, collaboration, integrated care, perinatal care, patient-reported outcome measures, value-based healthcare

## Abstract

**Introduction:**

Patient-reported outcome and experience measures (PROM and PREM) are used to guide individual care and quality improvement (QI). QI with patient-reported data is preferably organized around patients, which is challenging across organisations. We aimed to investigate network-broad learning for QI with outcome data.

**Methods:**

In three obstetric care networks using individual-level PROM/PREM, a learning strategy for cyclic QI based on aggregated outcome data was developed, implemented and evaluated. The strategy included clinical, patient-reported, and professional-reported data; together translated into cases for interprofessional discussion. This study’s data generation (including focus groups, surveys, observations) and analysis were guided by a theoretical model for network collaboration.

**Results:**

The learning sessions identified opportunities and actions to improve quality and continuity of perinatal care. Professionals valued the data (especially patient-reported) combined with in-dept interprofessional discussion. Main challenges were professionals’ time constraints, data infrastructure, and embedding improvement actions. Network-readiness for QI depended on trustful collaboration through connectivity and consensual leadership. Joint QI required information exchange and support including time and resources.

**Conclusions:**

Current fragmented healthcare organization poses barriers for network-broad QI with outcome data, but also offers opportunities for learning strategies. Furthermore, joint learning could improve collaboration to catalyse the journey towards integrated, value-based care.

## Introduction

The value-based healthcare strategy has shaped the development of healthcare systems towards a more person-centered and value-driven approach [1,2]. Defining value as outcomes for patients related to costs to deliver them, has aligned stakeholders to optimize value for patients [3]. Key-components currently adopted from this strategy include that professionals collaborate to organize care around patients’ needs and continuously measure outcomes that matter to those patients, such as functional status and quality of life [4,5]. Patient-reported outcomes and experience measures (respectively, PROM and PREM) are structured questionnaires that allow patients to report their health status and experiences with care [6]. In addition to clinical outcomes registry, PROM/PREM capture is therefore increasingly embedded in care systems to enable value-driven care at both the patient level: by discussing outcomes in clinical encounter to guide care decisions, and the population or patient group level: by evaluating aggregated outcomes for continuous quality improvement (QI) [7,8,9].

Although potentially promising, QI with aggregated PROM data of patient groups has been rarely described in the literature [10]. In value-based care research, examples of multidisciplinary QI with other outcomes data have been gathered: strategies included benchmarking, plan-do-study-act cycle, dashboards, and internal statistical analysis [10,11,12]. One of the main lessons from these projects, mostly conducted within organisations or single-provider networks, was that organisational readiness is needed for such an approach [4,11,13]. For many conditions, like frail elderly or pregnancy and childbirth, interorganizational collaboration in QI is needed to involve all professionals responsible for the outcomes of care [14]. In other words, patient-centred QI implies data collection, learning and innovating in integrated care networks, but what is needed to ensure network-readiness? Growing knowledge on network collaboration has emerged from many systems in transition to integrated care, including in perinatal care [15,16,17]. These transitions and accompanying research have offered valuable insights into collaboration processes across organizational boundaries and exposed barriers to be addressed at interprofessional level and at system level [16,18,19]. However, conditions and (learning) strategies for network-broad QI with outcome data are yet to be investigated.

This knowledge gap applies to present-day Dutch perinatal care as well (**Text box 1**), where collaboration in obstetric care networks (OCN) has increasingly integrated care around patients. We define integrated care as collaboration between care professionals and organizations providing medical and social care for pregnant women and mothers up to six months postpartum. Although joint, structural QI with patient-centred outcome data is considered an essential part of integrated care in their Care Standard as well, many OCN still struggle in practice to organize access to reliable data, joint learning strategies and follow-up of improvement actions [20,21]. In an implementation project guided by action research, three OCN aimed to both implement PROM/PREM assessment at individual level to guide patient care and use their aggregated data in network-broad QI cycles. Despite the complexity of simultaneous purposes, our pre-implementation research amongst key stakeholders suggested both levels could also reinforce each other [22]. Along PROM/PREM implementation in practice, this study focused on learning strategies for QI with outcome data in integrated care networks. Our aims were to 1) develop, implement and evaluate a learning strategy for patient-centred QI with outcome data in obstetric care networks and 2) explore and facilitate network collaboration factors that enable joint learning across organisational boundaries.

**Text box 1 Dutch perinatal care system**

Dutch perinatal care is provided multidisciplinary from two healthcare tiers: primary care by community midwives and maternity care organizations; and secondary/tertiary care by hospital employed care professionals. After this system became under pressure by relatively poor outcomes in 2004, care integration from all providers across the perinatal care continuum was considered one of the solutions to improve care continuity, perinatal health outcomes and even lifelong health of mother and child [23,24]. This potential solution was adopted by the Dutch government and the main parties within the sector [20,25]. Since then, hospitals, regional community midwife practices, maternity care and preventive child health organizations increasingly cooperate in local obstetric care networks (OCN) that aim to deliver high standard integrated care [21].

## Methods

### Design and framework

**Figure 1:**
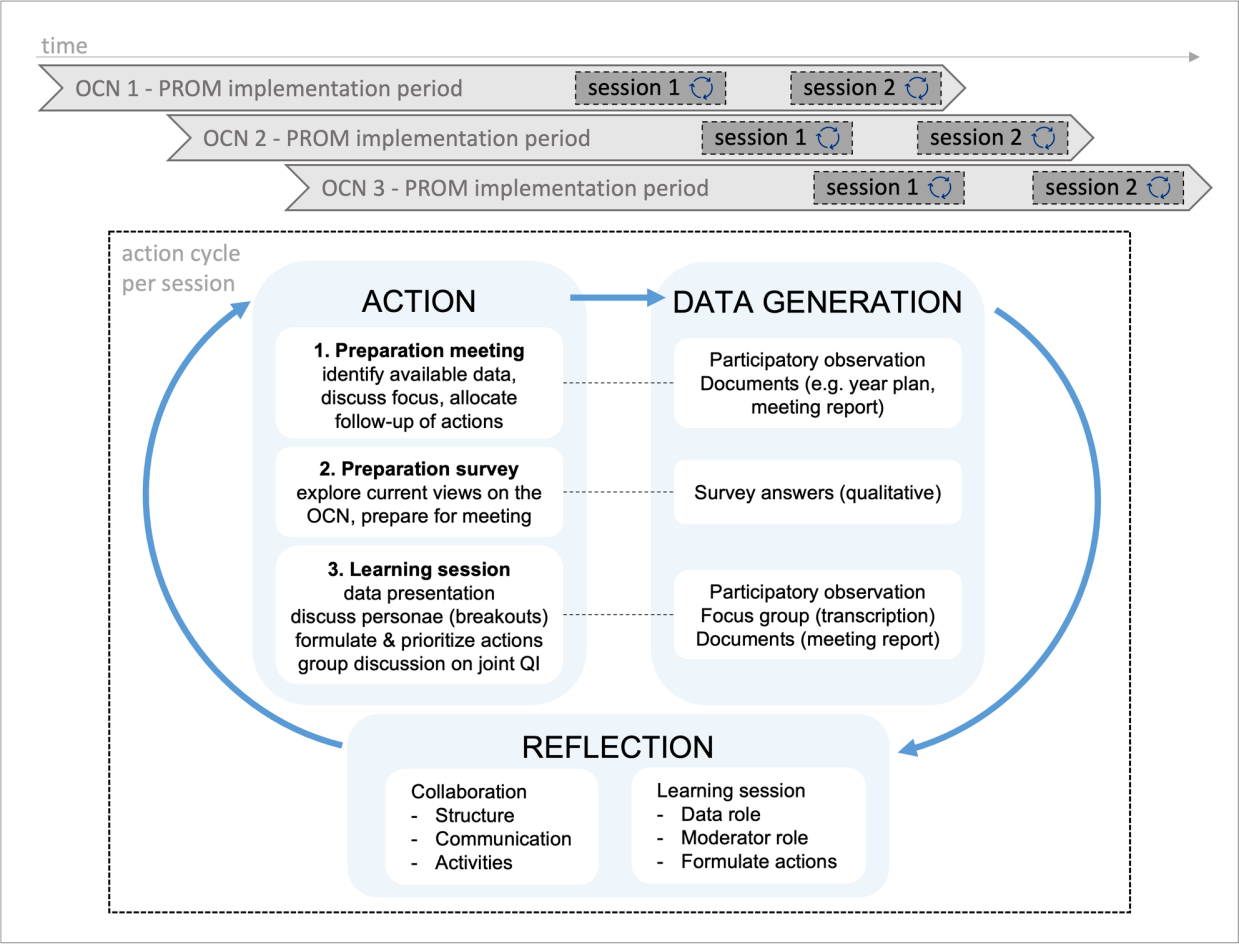
Study design: timeline and action research cycles per learning session. *OCN*, obstetric care network. *PROM*, patient-reported outcome measure. *QI*, quality improvement. *Implementation period* was 12 months in each OCN.

A qualitative observational study was conducted to investigate network-broad learning with outcome data. This study was embedded in an implementation project with the aim to implement PROM/PREM in routine practice of OCN, for which implementation analysis is described elsewhere [26], and subsequently in network-broad QI cycles based on aggregated results. A PROM/PREM set for perinatal care was used that was developed internationally, and tested recently in a national pilot [27,28]. The implementation process was guided by the principles of action research, an approach both to investigate practice change, whilst at the same time facilitating that change with researchers and participants collectively contributing to both aims [29]. This enables a broad understanding of complex practice changes and is done in a cyclic design of planning, action, data generation and reflection on data to plan subsequent actions. In this study, researchers and care professionals iteratively developed, implemented, reflected on, and adapted a learning strategy for QI with aggregated outcome data, concurrently gaining understanding of the complex conditions needed to learn and improve as care network. Each learning session corresponded with an action research cycle: to enable learning from previous cycles, the implementation project started in each OCN consecutively (Figure 1). As underlying theory, D’Amour and colleagues’ model for collaboration was used to determine the intensity of collaboration and link it to the ability to learn and improve as network [30]. Their model, consisting of four dimensions covering ten indicators, addresses both interprofessional and interorganisational collaboration and provides a typology to assess the intensity of collaboration via three levels per indicator (Table 1). This study was conducted between September 2019 and June 2022.

**Table 1:** Indicators for collaboration (based on the model and typology of D’Amour 2008).


CORE CONSTRUCT	INDICATOR	DESCRIPTION

Shared goals and vision	Shared Goals	The extent to which common goals have been formed and are supported by all collaborating partners.

	Client-centred orientation vs. other allegiances	The existence of asymmetric interests among partners and whether these are being expressed and negotiated.

Internalization	Mutual acquaintanceship	The presence of social conditions through which professionals get to know each other personally and professionally and create a sense of belonging to a group.

	Trust	Whether trust or uncertainty exists in each other’s competencies and ability to assume responsibilities, and whether this is grounded by previous experiences.

Governance	Centrality	Explicit and active involvement of central authorities with a well-defined strategic and political role to foster consensus and improve collaboration.

	Leadership	Type of leadership and balance of power in the collaboration: emergent or position-related, ad-hoc decisions or complete policy and shared or monopolistic.

	Support for innovation	The extent to which the organization draws on expertise needed to support complementary learning processes.

	Connectivity	Connection between parties through venues to discuss problems, find consensus and constructing bonds.

Formalization	Formalization tools	The degree of consensual agreements about roles and responsibilities: whether these are jointly defined and respected by all parties.

	Information exchange	The existence and appropriate use of an information infrastructure that meets care professionals’ needs for rapid, complete exchanges of information.


### Setting and participants

The implementation project in which this study was embedded, was initiated from a consortium of all OCN in the middle of the Netherlands (‘Geboortezorg Consortium Midden Nederland’, GCMN). The current Dutch perinatal care system is explicated in **Text box 1**. The project was carried out in three OCN, of which the hospital and several midwifery practices implemented PROM/PREM in their practice. The OCN characteristics are described in more detail in the implementation analysis [26]. In this study, regarding the learning strategy with aggregate outcomes, all care professionals working in these OCN could participate. Three levels of professionals’ participation could thus be defined: care professionals in the local project team (key participants), care professionals actively working with individual PROM/PREM results in practice (midwives and gynaecologists of practices participating in practice implementation), and other care professionals only joining the learning sessions with aggregated data (from non-participating practices or from other disciplines, e.g., maternity care assistants, nurses).

### Learning strategy

The purpose of the learning strategy was to support the OCN in setting up cyclical improvement of quality of care based on outcome data of their patient population. Its development was based on the IPEC (Interprofessional Education Collaborative) framework and a previous municipality project. Of the four core competencies of the IPEC framework required for interprofessional collaborative practice, the focus of the learning strategy was on competences in ‘Teamwork and Teambased practice’ (defined as *“Apply relationship-building values and the principles of team dynamics to perform effectively in different team roles to deliver and evaluate patient-centred care”*) [31]. The municipality project provided three years of experience in developing a learning strategy in which population data is used as basis to improve interprofessional collaboration between primary care, social care, and the municipality. The experiences from this project were translated to the OCN setting. In the sessions we aimed for first order learning from group level data, with an attempt to second order learning by analyzing different underlying reasons for particularly low or high scores per outcome domain of the set. This corresponds with the specific competences of the IPEC core competence aimed for.

The learning strategy consisted of three parts, which were reflected upon and adapted if needed in each action research cycle (Figure 1):

1. Preparation meeting: a one-hour meeting with a key-participant from each discipline to 1) prepare a session that matches current OCN goals and QI activities, and 2) engage key participants in session preparation to support embedding future improvement actions and sustainable learning cycles (even after the project). We aimed to discuss preliminary PROM/PREM results, identify additional data sources, choose important themes emerging from the data, adjust the preparation survey and session-invitation to local needs, and find possible follow-up structures for improvement actions formulated in the upcoming session.
2. Preparation survey: the goal was to 1) let participants think of the goal, topics, and expectations of the session beforehand, and 2) provide data for the session about the view of the professionals on current problems/challenges in their population.
3. Learning session: a three-hour session with five activities 1) icebreaking: exchange experiences with using PROM/PREM on a patient level and, in case of a recurrent session, reflect on intended actions of the previous session, 2) data presentation, 3) small, interprofessional group discussions about main themes from data along recognizable cases, 4) plenary discussion to share, choose and prioritize concrete improvement actions, 5) focus group discussion to reflect on collaboration conditions and needs for QI as network. Two sessions were planned per OCN: around 6-9 months and 9-12 months into the one-year implementation period.

The sessions were based on three types of data: clinical data, PROM/PREM data and care professional-reported data (via the preparation survey). Clinical data about pregnancy outcomes and population characteristics were retrieved from available data sources in OCNs, such as the Netherland’s Perinatal Care Registry (PeriNed) and via a national database on demographics per municipality (“waarstaatjegemeente.nl”). Together, the three types of data were used to create a shared understanding of the most important problems/challenges in the OCN’s population. These challenges were translated into individual, fictive personae reflecting recognizable cases in practice. Personae were discussed in interdisciplinary groups of 4-8 care professionals along a standard question format addressing positive and negative aspects of care for this persona. We aimed to achieve diversity in attending professional roles (i.e., minimum: a gynaecologist and midwife per persona, and a nurse, maternity care assistant and neonatologist per session). After re-joining again, each group summarized their conversation and concrete improvement actions were set, prioritized and allocated with all attendants. As final part of joint learning, the focus group offered space for collective reflections on collaboration conditions for QI as network and identify (local) needs for sustainable QI cycles. A session was summarized in a written document and a factsheet, to share the results across the OCN after a member check of the summary with the attending participants. Three researchers moderated the sessions together with the local project leader (a care professional).

### Data generation

A combination of qualitative methods was used to collect individual views of and generate group discussions with care professionals directly, as well as indirect via observations and documents (Figure 1):

- Qualitative survey: the preparation survey for participants to the learning sessions was used (Supplementary Table 1). It consisted of six open-ended questions and took 5-15 minutes to complete. Via a digital link, it was sent out with the session-invitation. Professionals who applied for the session received a reminder week beforehand.
- Focus group discussions: with care professionals attending the learning sessions who gave verbal informed consent. The topic guide based on D’Amour’s model concerned collaboration factors, current network-broad learning and conditions for outcome-based QI. One researcher (AD or AK) moderated the focus groups. Notes were taken by a second researcher and discussed afterwards (AK, AD and ML). Focus groups were transcribed ad verbatim.
- Participatory observation: three researchers (AD, AK, ML) performed participatory observations at the preparation meetings and learning sessions, supervised by a senior action researcher (BP). Notes were taken about network collaboration, roles of and interaction between professionals (and researchers), and elements of the learning sessions. Afterwards, the researchers reflected upon the notes and saved them in a logbook.
- Documents: written documents regarding OCN collaboration and learning process (e.g., vision document, year plan, meeting reports) were saved for analysis.

### Data analysis and reflection

During the study period, researchers and participants iteratively developed and executed learning activities, generated data on their experiences and reflected on those data, which shaped the learning strategy and subsequent data generation. For example, reflections in dialogues between researchers and participants were used to adapt the topic guide for focus group discussion to address collaboration aspects important in context and time. Also, participants reflected on (a summary of) survey results, observations and focus group data, during respectively the session, the focus group, via the session summary and meeting reports. This way, participants contributed to joint reflection, interpretation of data, and planning further actions in practice, while member checking research data at the same time. A structured reflection journal was kept (by AD or MR) and doubts, unexpected events, or “arresting moments” were discussed every two weeks (with BP) to strengthen this process. Eventually, qualitative data from all sources (i.e., survey answers, focus group transcriptions, observations, logbook and documents) were aggregated in one document per OCN and thematically analysed by two researchers (AD, AK) conform QUAGOL guidelines, using a combined deductive and inductive approach [32]. Guided by D’Amour’s model, this process included the following steps: data familiarization, initial coding (two documents by both researchers), discussing differences to reach consensus and develop a mature coding scheme, further coding of all data, summarizing main themes per document, charting and mapping all coded fragments, and interpretating data. The development of the coding scheme and interpretation of results was discussed with three senior researchers to reach consensus (BP, MR, MB). We used Microsoft Word for coding and Microsoft Excel (version 16.64) for mapping and analysis: a systematic way to structure qualitative data as described by Ose et al. [33].

## Results

**Figure 2:**
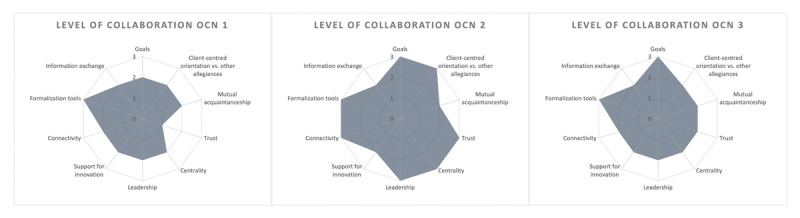
Collaboration levels of participating OCN. These Kiviat graphs map the collaboration per OCN: a score of 1 to 3 is assigned to each of the 10 indicators depending on the level of achievement of the indicator in the OCN [30]. *OCN*, obstetric care network.

Across the three OCN, five learning sessions were organized, four of which took place online because of the COVID pandemic. One OCN organized only one of two indented sessions: after stopping PROM/PREM capture after the one-year implementation period mainly because of IT issues, this OCN wanted to invest first in solving IT issues and improving collaboration before putting their time and efforts in a second session. On average 17 professionals attended the sessions, representing four to six different disciplines and four to seven organizations (Table 2). The preparation meeting before each session was attended by mean four care professionals (range 2-8). In total 60 preparation surveys were returned. Five focus groups were held, one in each session, with a total of 78 care professionals participating. Overall collaboration levels across the study period varied per OCN (Figure 2), of which intermediate assessments were used to prepare meetings and reflected on with participants. After merging all data sources, thematic analysis resulted in an overall evaluation of the learning strategy and collaboration factors affecting network-board learning.

**Table 2:** Characteristics of learning sessions.


	SESSION 1	SESSION 2	SESSION 3	SESSION 4	SESSION 5	TOTAL	TOTAL UNIQUE

**Region**	OCN1	OCN2	OCN2	OCN3	OCN3		

**Location**	online	online	live	online	online		

**Participants**	16	25	11	16	19	87	70

community midwife	9	11	5	8	10	43	33

hospital midwife	1	6	3	3	2	15	12

obstetrician/gynaecologist	2	2	2	2	4	12	9

obstetric resident		6		1	2	9	9

youth care professional	1					1	1

obstetric nurse			1	2	1	4	3

maternity care	2					2	2

neonatologist/paediatrician	1					1	1


*OCN*, obstetric care network.

### Learning strategy evaluation

Iterative reflection and adaption of the learning strategy with researchers and care professionals resulted in main challenges and successful elements, for which illustrative quotes are listed in **Text box 2**.

**Text box 2 Supportive quotes learning strategy evaluation**

Successful elements of strategy

**Table:** 

Interdisciplinary discussion	**Q1** Clinical midwife OCN2, focus group – “*It [the subgroup discussion] is very small and compact, everyone brings their expertise from their own profession. I also think that it goes very harmonious. And as a result, such follow-up visit [improvement actions], that it arises in both groups: that wouldn’t emerge in a regular meeting.”*
Interdisciplinary discussion and data insight	**Q2** Clinical midwife OCN3, focus group – *“In the past, we did look regularly at clinical data and actions were taken. [..] But then, I agree with [a gynaecologist], in a meeting like this one, where you can also discuss data more in-dept and concrete with each other [..] then I think you will be able to realize improvements and adjustments much better together.”*


Challenges for learning strategy

**Table:** 

Follow up of actions	**Q3** Gynaecologist OCN3, observation of preparation meeting – *“GYN states they were still habituating in the first session and must seek as OCN who picks up the actions. The actions of first session have been submitted to the OCN board but have remained there.”*
Engage all disciplines	**Q4** Clinical midwife OCN2, focus group – *“The intention is that we will involve nurses and the maternity caregivers much more in the OCN, and inform them much more about what it all means and what topics are at stake. And that they also have input on that.”*

#### Enablers in the learning strategy

Successful elements included insight in (patient-reported) data, interprofessional discussion along personae, plenary prioritizing, and joint reflection.

– Professionals in all OCN were enthused by the insight in *data directly from their patients*. Clinical data about their network had been presented before, and sometimes discussed for quality performance as well, but the combination with patient-reported data provided a more complete view of their patients’ wellbeing and experiences (e.g., breastfeeding, shared decision making). Session participants emphasized that data presentation should be short and concise and highlight both positive and negative outcomes.
– *The translation of data into personae* reflecting most important challenges was praised in participants’ reflections and enabled a conversation about the provided care for that persona in all observations, while limiting the discussion about the quality of the data themselves (e.g., casemix factors, representativeness). Also, reflected in the sessions’ output, professionals were in the lead which part of the persona was most important, i.e., the main challenge they encountered in practice for this persona.
– Session participants agreed on the value of *interprofessional discussion* in small subgroups about improvement opportunities in practice. Based on survey answers and focus group opinions, these discussions were most valuable if a diverse range of professionals joined and shared various perspectives. Regardless of the exact topic, participants in subgroups were observed to share expertise, find consensus, and use each other’s qualities or initiatives.
– After subgroup discussions, *the plenary conversation* was found essential to prioritize and allocate improvement actions to individuals or existent working groups, which required time and active moderation. Sometimes, multiple subgroups (i.e., that had discussed different personae) shared similar improvement actions here, which gave participants a feeling of consensus and urgency.
– Iterative *joint reflection* on sessions and local collaboration, both in the preparation meeting and collective focus group, was noted to enhance professionals’ ownership over the QI process and adjust it to contextual factors and priorities. In the preparation meeting, key participants incorporated current OCN goals in the preparation survey, discussed how to engage all disciplines, and set priorities and goals for the session. Also, specific collaboration themes that arose in preparation meetings could be incorporated in focus group statements, which helped to create collective discussion and form consensus on these collaboration themes.

#### Barriers in the learning strategy

Elements that posed challenges included care professionals’ time constraints, data infrastructure, engagement of all disciplines, and formulating actions and their follow-up.

– *Professionals’ time constraints* were one of the main reasons for absence, interrupted meetings or partial attendance, partly due to the acute nature and irregular hours of perinatal care. Although they felt learning is part of their normal job, all participants conformed that preparing and attending network-level learning or QI always came on top of regular working hours. Besides demanding patient care, some focus group participants emphasized that personal priorities and the OCN culture influenced available time and efforts for collaboration and joint QI as well.
– *Data infrastructure*. In all OCN, participants and researchers experienced difficulties to gain valid, real-time data. Network-broad clinical data were often outdated or unreliable due to registration issues. Moreover, little PROM/PREM data were available due to IT challenges that persisted during the implementation projects and couldn’t be merged directly with clinical data. Also, data preparation (access available sources, analyse, visualise) took much time and had to be conducted largely by the researchers. In the OCN with a quality manager, this process was easier and resulted in more valuable data.
– *Follow-up of improvement actions* differed in success per session, and joint reflections pointed out two aspects: OCN collaboration structure and nature of improvement actions. A clear and active collaboration structure to set priorities and divide responsibilities was considered helpful to allocate the actions directly to the right persons or existing working groups. Researchers noticed that a confined range of improvement actions came up (e.g., practical, direct actions, education), and that a broader action repertoire could enhance finding suitable and effective solutions.
– Several participants noted that *engaging professionals* not working with PROM/PREM in practice had added value but was harder. Moreover, existing gaps between professions or organisations were considered difficult to bridge. In several focus groups, participants expressed a need to increase involvement of nurses and maternity care in their OCN. If attending a session, often a manager came, who could contribute less to a persona discussion because of little practice experience.

Based on reflections, improvements made to the sessions in general included a more concise data presentation, a longer plenary end to prioritize and allocate actions, a list of possible action levels to broaden the range of thinking, and adjustments to the persona format to navigate the subgroup discussions better. After the first two sessions (online), the topic guide for focus group discussion was transformed by the researchers into statements to provoke discussion and engagement of all participants. In the next sessions, these statements could be adapted easier to collaboration topics important in local context and time, based on reflection in dialogue between researchers and care professionals.

### Collaboration factors affecting joint learning

Thematic analysis of collaboration in the networks and the influence on the ability to learn and improve as network was summarized in Table 3 along the indicators of D’Amour. Below we elaborate on the indicators that contributed mostly to the ability to learn and improve as a network, for which illustrative quotes are listed in **Text box 3**.

**Table 3:** Framework analysis of network collaboration and learning along D’Amour model.


INDICATORS OF COLLABORATION	THEMATIC ANALYSIS(SUMMARY WITH SUBTHEMES IN BOLD)

**Shared goals**	All OCN had a shared patient-centred goal: best possible outcomes and continuity of care. **Year plans** to reach their goal were formalized in OCNs to various extent, and in each organisation (e.g., a hospital) separately. This could lead to **fragmentation**, dependent on the network’s governance. For learning, shared goals were important, but should be **concise and focused** (not too many or too broad).

**Client-centred orientation vs. other allegiances**	All OCN **centred patients** in their vision, but it differed to what extent **other allegiances** overruled that (e.g., professional autonomy, financial structures). Also, professionals had **divergent views** on what benefits patients most. All OCN wished to involve **patient views** in learning/improving, especially when selecting or evaluating new initiatives, but struggled to do so (see information exchange).

**Mutual acquaintanceship**	In all OCN, professionals stated that knowing each other and meeting regularly were of greatest importance for good collaboration. When feeling part of the OCN was **limited to a few key participants**, the network was depended on the same people who were very motivated but needed broader engagement for results. Participants identified **stakeholders** needed for learning as all professionals involved in care and patients themselves. Yet in all OCN, engaging **nurses and maternity care assistants** in network activities was challenging. Knowing what occurs in the OCN and experiencing their valid contribution could help them become more involved.

**Trust**	Care professionals stressed trust as most important, the **base**, for collaboration and joint learning/improving. Important for trust were **respect** for divergent opinions and **acknowledgement** for qualities across disciplines. All OCN had built some level of trust from fragile to grounded, but differed in whether that was **maintained** over time, and how broadly it was **shared** across professionals. Trust was determinative for working pleasure/atmosphere perceived by care professionals and was mostly influenced by the level of connectivity and mutual acquaintanceship.

**Centrality**	Centrality was not often chosen or stated by care professionals as important factor, but indirectly they mentioned that improvement initiatives should **not overlap**, and **consensus** and **clarity** existed on goals and plans of the OCN. In OCN with an inactive central body (for several reasons, see leadership), initiatives were **fragmented** and proceeded slow as it was harder to **allocate actions**.

**Leadership**	Leadership varied across the OCNs and noticeably influenced the ability to learn and innovate together. If leadership patterns were observed more **fragmented** across organizations, ad-hoc decisions and **unclarity** where decisions should be made often resulted in **top-down** decisions eventually – which were then less likely to be accepted by professionals in practice. Leadership **structures** were still developing, and professionals noted that its changes affected their connectivity and mutual acquaintanceship.

**Support for innovation**	OCNs experienced little support not necessarily in a lack of expertise, but in **time** (workload, priorities) and **resources** (data availability and analysis, digital support). In two OCNs the working group for quality improvement was inactive or even absent. In OCN2 it was stated they ‘bought time for innovation’ to some extent by allocating administrative support and a quality manager for the OCN, possible via a **joint reimbursement** structure. Care professionals indicated that learning and QI felt as a **normal part** of their professional role. On a personal level, they **learn and improve every day** during work, but network level learning or QI always comes **on top of their normal job**, often in late hours as patient care comes first. For care professionals, learning/improving was stated to be **easier within organisations than in a network** (challenging to engage all stakeholders) but they expect most **value** for patients from a network approach.

**Connectivity**	Connectivity was highly important for collaboration and innovation, both from professional’s views as from observations. First, regular venues for discussion were essential to **form consensus or accept differences** in vision and **make use of each other’s expertise**. Second, connectivity in the way that professionals knew from each other **what they were working on** and what their **level of commitment** was. Both contributed positively to trust between OCN professionals, their sense of belonging (mutual acquaintanceship) and ability to work simultaneously instead of fragmented.

**Formalization tools**	All OCN experienced positive results from their joint formalization tools (e.g., joint protocols, shared care pathways, standard collaboration partners). In the past years, this has been their **primary focus** to improve quality and **continuity** of care. While many survey respondents expressed a need for more formalization, others emphasized that attention should remain for **patient’s values and individual choices** in care paths. In QI, formalization was considered and observed as a tool to embed actions in practice.

**Information exchange**	As almost all organisations worked in different EHRs, each OCN faced problems with information exchange (i.e., e-mail, fax, on paper) and mandated a **shared or connected EHR** to enable easier communication in practice and better access to aggregated data for learning and QI. **Reliable data** were stated essential for QI and learning but are hard to access or require much effort. Moreover, patient-reported data are not accessible at network level at all (except during the implementation period), making it difficult to **involve patient** views in learning and QI.


*OCN*, obstetric care network. *EHR*, electronic health record. *AR*, action research. *QI*, Quality Improvement.

**Text box 3 Supportive quotes collaboration factors affecting joint learning**

Baseline collaboration needed

**Table:** 

Collaboration baseline	**Q5** Clinical midwife OCN1, focus group – *“I think it’s until you have your act together as OCN that it will be fun to look at those outcome data together.”*
Trust and other allegiances	**Q6** Obstetric resident OCN2, preparation survey (item 6: when is the session successful for you?) – *“If we work together on outcomes without restrictions in trust or finances/autonomy: ‘what is best for the pregnant woman?’”*
Connectivity vs leadership	**Q7** Gynaecologist OCN3, focus group – “*I also think that what she said [statement of clinical midwife] is a somewhat broader endorsed dissatisfaction. That, with the implementation of the new [leadership] structure, too much goes via mandate or too much goes via a limited number of people. That the joint meetings [in the past] really added something.”*


Conditions for joint learning

**Table:** 

Information exchange	**Q8** Obstetric resident OCN3, preparation survey (item 2: what do you need as professional to address these themes?) – *“A joint EPD, this also ensures more efficiency and less chance of errors, because then we don’t have to retype anything.”*
Support for innovation: joint reimbursement	**Q9** Gynaecologist OCN2, focus group – *“But we can buy that time by being an integrated care organization: by having a quality officer, having secretarial support, having a manager. We buy off all kinds of things, so to speak, so that we have time for learning and improving”*

Before learning and improving together, a collaboration based on *trust* was explicitly stated essential in focus groups and survey answers and reached most noticeably through *connectivity* and *leadership*. In the sessions, professionals unanimously agreed that *trust* was the base of collaboration, including respect for divergent opinions or visions and acknowledgement for different qualities per profession. Although all OCN expressed a shared patient-centred goal and vision formalized in their plans, professionals described variation in the extent to which *connectivity* was present to discuss differences (in opinions, visions, other allegiances), find consensus, and share commitments to reach those goals. If connectivity decreased, or was confined to a small number of professionals, increased fragmentation was described and observed on several collaboration aspects, such as goals, formalization tools and decision-making. Arising from joint reflections in group discussions and observations, collective *leadership* that invested actively in broad *connectivity* and gave regular feedback could improve *trust* in collaboration on all these aspects, whereas ad-hoc and fragmented decisions could even cause distrust. For example, top-down decisions made in a single organization surprised care professionals in (other) practice(s) and were less likely to be accepted by professionals in practice, both affecting the level of trust negatively.

When a base for collaboration was present in an OCN, their ability to learn and improve together was influenced mostly by *information exchange, support for innovation*, and *centrality*. Current *information exchange* posed a barrier in all OCN: each of them searched better access to aggregated data – especially patient-reported data. An integrated approach to innovation was believed most valuable for patients. Yet important barriers classified in *support for innovation* were time constraints for care professionals and, along that, financial support for joint innovation (e.g., participation in working groups, performing leadership roles, data analysis and visualisation). Most tasks were thus performed voluntarily, making these efforts vulnerable to professionals’ individual motivation and priorities for QI. In OCN2, a quality manager and administrative support could be allocated from their joint reimbursement structure, which supported them significantly in joint QI. When the strategic and political roles within an OCN (*centrality*) were clear to all professionals and carried out actively, new initiatives were easier to allocate and follow-up.

## Discussion

In three OCN using PROM/PREM for individual care, a network-broad learning strategy was developed with the aim to set up cyclic QI with aggregated data of their population using clinical, patient-reported and professional reported data. Guided by an action research approach, the learning strategy was implemented, evaluated and adapted simultaneously to gaining knowledge of network collaboration conditions that enable joint learning. In all OCNs, the learning strategy created a venue for in-dept interprofessional discussion and helped to identify improvement opportunities for quality and continuity of care across the perinatal care continuum. Main challenges were professionals’ time constraints, follow-up of improvement actions and data accessibility. The significant differences found in network collaboration affected their ability to activate joint learning and improvement cycles. First, readiness of a network to learn together depended on a baseline collaboration with trust, reached most noticeably through connectivity and consensual leadership. Second, sustainable joint learning and improvement cycles required information exchange and support for innovation in terms of time, data, and resources.

In line with literature, collaboration was only possible when grounded trust was present, thus fundamental for joint learning too [30,34]. Trust between maternity care professionals is an area of tension historically, originating from several factors including professional autonomy, financial incentives, and divergent paradigms on the physiology of pregnancy and birth [35,36]. In our project, these tensions emerged as well to some extent in all OCNs striving for integrated care, but important variations in trust were found between OCNs (e.g., the degree of trust, how broadly shared, at practice level and/or at managerial level). A crucial factor for whether OCN had built and maintained trust appeared the level of connectivity to discuss issues, form consensus, and build mutual accountability in relations. Here, an important role for leadership emerged to foster connectivity, participatory decision-making, and clear communication about decisions to, subsequently, build trust. Networks with collective, consensual leadership expressed more connectivity compared to top-down power relations or fragmented leadership patterns. This resonates with previous reflections on leadership and power dynamics in integrated care, that state a need to reflect on power as dispersed and negotiated throughout the network and its actors, instead of power as bidimensional; and a need for collective leadership to build trust, distribute accountability, power, and funding across organisational boundaries [37,38]. Thus, to build and maintain trust throughout the journey towards integrated care, connectivity built in daily practice between professionals must be supported by leaders, who have the time, resources, and drive to organize common ground to manage conflict and form consensual decisions on a continuous basis.

A main barrier for collaboration and joint learning across the networks was a lack of time, and underneath that, the resources to make time. Although mentioned as external factors influencing collaboration, resources and financial constraints were not included as internal collaboration indicators by D’Amour [30]. In contrast to our findings, where internal structures in network governance (i.e., joint reimbursement agreements) affected the availability of time and resources for collaboration and were interconnected with trust and shared goals as well. For instance, the level of trust and shared goals influences the decision to become an integrated organisation with a juridical entity for joint reimbursement agreements, which, in turn, creates opportunities to further collaboration with shared resources and responsibilities, decreasing other allegiances than client-centeredness. Therefore, time, resources and financial agreements reflect collaboration and should be considered when evaluating and improving network collaboration as part of governance. Still, as emphasized by others as well [16,17], external system-level changes are required that address the structural barriers for collaboration to enable possibilities for joint reimbursement agreements in networks that feel ready.

In the learning strategy, the outcome data feedback was valued as it helped to identify opportunities to improve care and stimulated care professionals in their willingness for QI. At the same time, the available data for the sessions were far from optimal and their gathering and analysis (especially patient-reported; organized temporarily during the implementation projects) took much time and efforts. Noticeably, the learning strategy facilitated a shift from a discussion about data (or their quality) towards content of care by translating the main themes emerging from the data into personae, which were then discussed along a structured format. Even with better-quality data, this strategy might help to focus on content of care, as case-mix factors and validity of the data (i.e., whether the data truly measure value of care) will always be subject for discussion to some extent. Another benefit of this data-to-persona strategy was that professionals were in the lead of important aspects of care that needed change for a persona. As such a strategy depends on professionals’ capability to observe, interpret and reflect broadly on possible solutions to produce effective actions, future (action) research could explore how knowledge on learning to learn (third order learning) could benefit the VBHC strategy [39]. Empowerment of professionals in learning can contribute to workforce development, working culture and their ownership of QI initiatives [40,41]. Thereby supporting to rebalance the reinforcing relation between bottom-up initiatives and top-down directives needed to implement integrated care [42]. Although the data-to-case strategy provided short-term opportunities for joint learning, sustainable resources for (patient-reported) data capture are needed to empower professionals further in QI, such as digital questionnaire tools and infrastructures to merge clinical and patient-reported data across providers.

**Figure 3:**
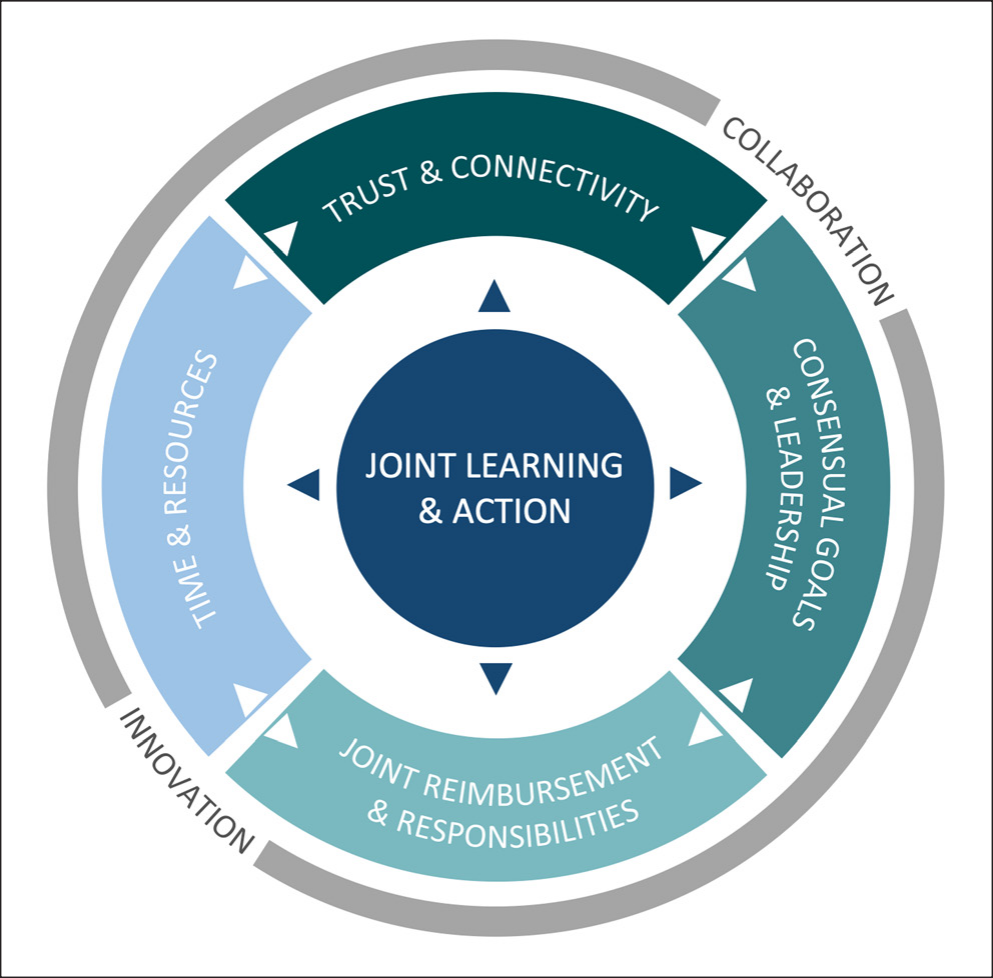
Joint learning in relation to collaboration and innovation in care networks.

In short, the structured subgroup discussions linked to the themes but disconnected from the direct data created a venue for discussion that, although not directly measured, presumably contributed positively to connectivity and trust – especially if some of that was already present. So, not only collaboration factors affected the ability to learn together but, reversed, learning activities seemed to influence collaboration: creating a cyclic effect between collaboration and innovation visualized in Figure 3. A similar effect was described in a study of multidisciplinary teams, stating that joint actions – more than vision or strategy – have the potential to catalyse integrated care [43]. A challenge for collaboration and learning encountered in our project was how to engage all disciplines needed and, if engaged, a broader group than just key stakeholders. Nurses and maternity care, for example, were considered important stakeholders but were less connected to the OCN in general. Our findings do provide a direction for improvement via joint learning and action.

### Strengths and Limitations

The use of D’Amour’s typology strengthened our analyses and understanding of collaboration mechanisms and the way they influence joint learning. The combination of focus groups, open-ended surveys, and observations enabled data triangulation from multiple sources and various perspectives. Although logbook data were not member-checked, all findings were verified in the process of joint reflection and the interaction between professionals and researchers during action cycles. Still caution is needed with generalizability, as researchers and participants were focused on applying the findings and improving the strategy to the local context. The usability and future sustainability of the learning strategy was enhanced by care professionals’ participation in actions and reflections on the needs for collaboration and learning. When implementing the strategy in other settings, this adaptive and reflexive design should be adopted as well to match local needs. With three different network settings participating consecutively, the iterative action research process contributed to improve the sessions in general, reach data saturation in research activities, and adapt to the exceptional circumstances during the COVID pandemic. Still, the COVID-19 pandemic has influenced our findings not only because online contact limited interprofessional interaction in group discussions, but also because other joint activities were on a lower level and professionals’ workload was extra high. Although unfortunate in terms of data to evaluate and improve the strategy, one session not being carried out provided insight in the conditions needed to carry out collective learning at the same time. Ideally, patients would have participated in the learning strategy too, but this was chosen not to organize in this stage yet. As individual answers were discussed in clinic, patient interpretation of PROM/PREM data was ensured: these experiences were brought along by professionals that participated in the learning session. In future sessions, active patient involvement in the data interpretation part, the persona discussions and/or the preparation session can be piloted and evaluated.

### Practice implications

The lessons learned in this study lead to both short term practical advice to set up QI with outcome data feasible in current OCN practice with shortcomings in data and resources, supported by long-term strategies to improve conditions for joint learning in terms of data and collaboration. During the project, the sessions were largely dependent of the researchers [26], but the project leaders were actively involved in each step and the development of a manual for OCN to feasibly organize these sessions with currently available data sources and time. In this manual, short time implications to organize learning strategies for QI with outcome data are 1) combine available data sources and use them pragmatically (e.g., personae, question format) to generate meaningful discussions; 2) work in an iterative design to adjust to local collaboration and existent QI processes; 3) invite all disciplines and organizations as multidisciplinary discussions could improve the value of learning sessions and the connectivity across the network; 4) embed new learning strategies in policies (e.g., Standard of Care, training, accreditation) with sufficient support, to reduce the burden of QI initiatives on professionals and create short-term external incentive. Long term implications for network collaboration and learning include 1) invest in network-broad data infrastructures including patient-reported data [44,45]; 2) explore joint reimbursement structures to enable sustainable joint learning and follow-up of actions; 3) create sustainable, collective leadership structures that foster connectivity, joint actions and thereby trust.

### Conclusions

Before integrated care and joint quality improvement based on (patient-reported) data will become normal practice, important challenges exist in current fragmented healthcare organization on system-level, data-level and professional-level. Despite those barriers, this study exposed ways to organize collective learning for QI in present practice. Network-broad learning and improvement based on outcome data has the potential to improve continuity of care, working pleasure, and eventually patient outcomes and experiences. This action research project resulted in a learning strategy for QI in perinatal care networks, adapted to care professionals needs and, with a cyclic and participatory approach, transferable to other integrated care networks as well. Our analysis of network collaboration contributes to the understanding of complex processes towards integrated care with patient-centred care improvement, translated into concrete implications for practice.

## Appendix

**Supplementary Table 1:** Preparation survey for learning sessions. *OCN*, obstetric care network. All questions had open ended answer fields. In each preparation meeting, this survey was discussed with key participants in the OCN and adapted slightly to fit the regions current goals for quality improvement.

Introduction: All attendants of the learning sessions are asked to complete these preparation questions. For you as preparation to the session and for us to organize a valuable session fitted to your OCN and patient population.

Baseline: For which organisation do you work? What is your function?

Survey: In the learning session we will work on the current goals in the OCN [adapted per session and region]. Which of these themes should we address as OCN first? What do you need as professional to address these themes? Do you miss any issues/themes that we could improve as OCN? For example, specific patient groups, outcomes, or experiences. According to you, which activities (initiatives/agreements/collaboration) in the OCN have yielded most value (client/patient; care professional; financially)? What should we stop doing? When is the session successful for you?
